# *RFX6* maturity-onset diabetes of the young: clinical considerations and novel use of tirzepatide

**DOI:** 10.1530/EDM-25-0142

**Published:** 2026-01-22

**Authors:** Prethivan Pillai Gopalakrishnan, Amit Banerjee, Rachael Milne, Rebecca Smith, Steven John McNulty, Sumudu Bujawansa, Ram Prakash Narayanan

**Affiliations:** Department of Diabetes and Endocrinology, St Helens Hospital, Prescot, United Kingdom

**Keywords:** RFX6-MODY, pregnancy, tirzepatide

## Abstract

**Summary:**

*RFX6* maturity-onset diabetes of the young (*RFX6*-MODY) is a relatively new MODY subtype, with limited guidance on management, particularly in pregnancy. We report the clinical features and management of two female patients with *RFX6*-MODY and their progression during and post-pregnancy. These patients were diagnosed with type 2 diabetes mellitus (DM) at ages 13 and 19 years, initially managed on dietary modification alone. They were subsequently diagnosed with *RFX6*-MODY during pregnancy following calculation of MODY probability. Both required insulin during pregnancy and delivered healthy babies at 38 weeks. Three months post-delivery, tirzepatide was started for one of our patients and she has shown significant glycaemic improvement and weight loss. To our knowledge, this is the first reported use of tirzepatide in *RFX6*-MODY.

**Learning points:**

- *RFX6*-MODY may present at a much earlier age than previously reported in the literature.
- Many patients with *RFX6*-MODY do not appear to require insulin at diagnosis.
- Tirzepatide may be a beneficial therapeutic option for managing patients with *RFX6*-MODY who have adequate β-cell function.
- Pregnancy management in patients with *RFX6*-MODY is similar to type 2 DM, although higher insulin doses may be required.

## Background

MODY is caused by a single gene mutation but has a heterogeneous phenotype. The prevalence of pathogenic MODY variants in the UK is approximately 1 in 1,052 individuals, accounting for about 1.5% of diabetes cases diagnosed before the age of 40 (1). It usually manifests before the age of 25 years in an autosomal dominant pattern and lacks pancreatic antibodies. *RFX6*-MODY is a relatively new MODY subtype, which was first reported in 2014 (2). The regulatory factor X6 (*RFX6*) encoding gene is located on chromosome 6q22.2. *RFX6* is a transcription factor expressed in the pancreatic islet cells, proximal small intestine and colon. *RFX6* is important in the differentiation of pancreatic beta cells and production of insulin (3). It is also associated with glucose-dependent insulinotropic polypeptide (GIP) expression and secretion from the enteroendocrine K cells at duodenum and jejunum (4). It is important to acknowledge that new variants of MODY may have different phenotypes. Therefore, we describe two patients with *RFX6*-MODY and their management including perinatal medical consideration. Notably, this case series includes the first reported use of tirzepatide in *RFX6*-MODY, highlighting a potential therapeutic option for this MODY subtype.

## Case presentation

### Case 1

**Figure 1:**
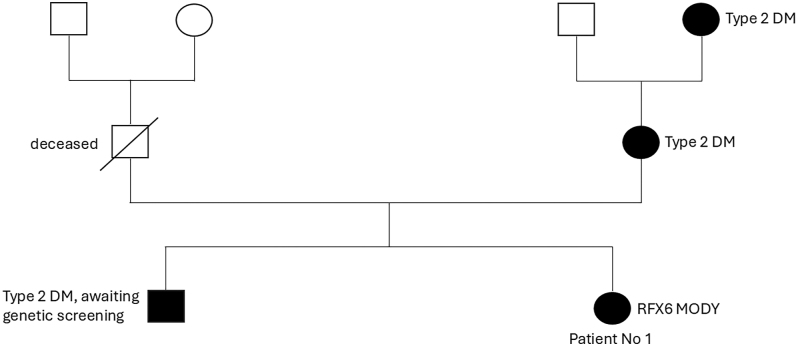
Family pedigree of patient 1. The squares represent males, and the circles represent females. The shaded symbols indicate individuals with diabetes. A diagonal line through a symbol denotes a deceased individual.

A woman with a family history of diabetes in her mother and brother (Fig. 1) was diagnosed with type 2 DM when she was 19 years of age. At diagnosis, her body mass index (BMI) was 31.6 kg/m^2^, and her HbA1c improved from 81 to 49 mmol/mol with dietary modification. She required metformin and insulin during her first pregnancy, but metformin was discontinued due to gastrointestinal intolerance. Following her second pregnancy, dapagliflozin was initiated for its mild weight-loss benefit, but poorly tolerated. She declined insulin and was therefore managed with pioglitazone. She presented to our clinic during her third pregnancy, at the age of 31 years.

### Case 2

**Figure 2:**
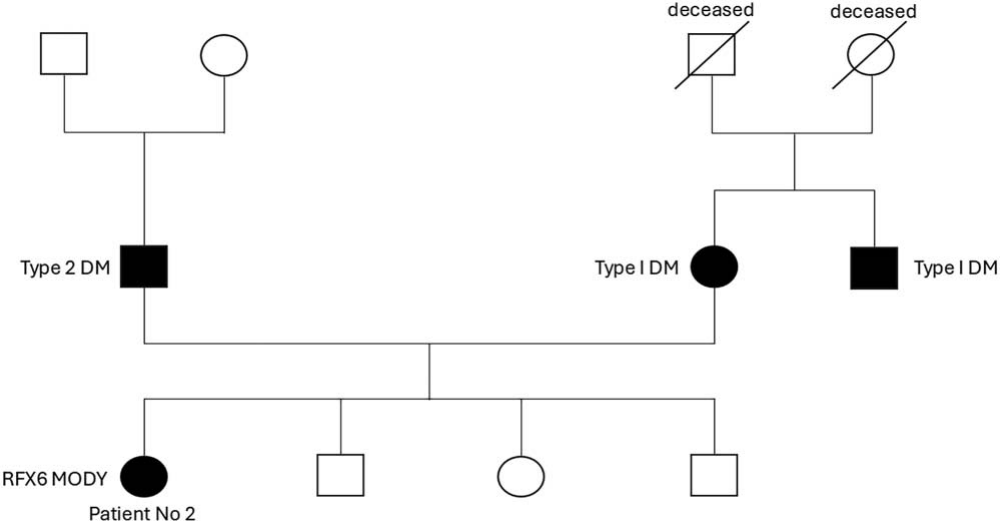
Family pedigree of patient 2. The squares represent males, and the circles represent females. The shaded symbols indicate individuals with diabetes. A diagonal line through a symbol denotes a deceased individual.

A woman with a family history of diabetes in both parents (Fig. 2) was diagnosed with type 2 DM when she was 13 years of age. She presented to the diabetes antenatal clinic at the age of 23 years and 8 weeks of gestation, with an HbA1c of 54 mmol/mol and a BMI of 43.5 kg/m^2^. She had been started on metformin two weeks prior to her first antenatal appointment, having not been on any diabetes medication previously.

## Investigation

### Case 1

During her first antenatal diabetes appointment, at 6 weeks of gestation, her HbA1c was 55 mmol/mol. She had a MODY probability score of 45% using the Exeter University MODY Probability Calculator (MPC), due to the early onset of diabetes and a positive family history. Hence, genetic testing was sent, which confirmed *RFX6*-MODY (NM_173560.4:c.224-12A>Gp). Circulating GIP levels were not measured. Her C-peptide level was 483 pmol/L (reference range: 190–990 pmol/L) with a blood glucose level of 4.0 mmol/L. Glutamic acid decarboxylase 65 (GAD65), islet tyrosine phosphatase 2 (IA2) and zinc transporter 8 (ZnT8) antibodies were negative. She has no evidence of microvascular or macrovascular diabetes complications.

### Case 2

During her first antenatal diabetes appointment, at 8 weeks of gestation, her HbA1c was 54 mmol/mol. Her C-peptide level was 599 pmol/L, with a blood glucose level of 7 mmol/L. GAD65, IA2 and ZnT8 antibodies were negative. Her MODY probability score was 62% using the MPC, in view of the early onset of diabetes and a positive family history. This prompted genetic testing, which confirmed *RFX6*-MODY (NM_173560.4:c.866del,p.His289LeufsTer55). Circulating GIP levels were not measured. She has no evidence of microvascular or macrovascular diabetes complications.

## Treatment

### Case 1

**Figure 3:**
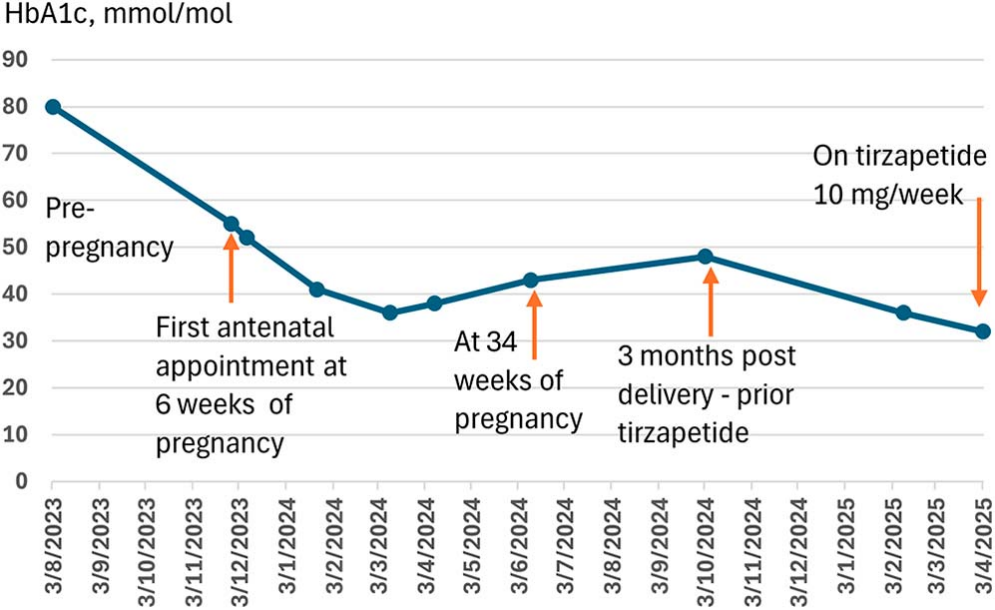
HbA1c trend in patient 1. The graph displays serial HbA1c measurements for patient 1, showing glycaemic control before, during and after pregnancy.

She was commenced on Lispro insulin (Humalog^®^) 8 units with meals and isophane insulin (Humulin I^®^) 26 units daily, alongside dietetic input, and was reviewed fortnightly in the antenatal clinic. At 36 weeks of gestation, ultrasound assessment showed a fetal abdominal circumference above the 97th centile. By this time, her insulin requirements had increased to Lispro 28 units with meals and isophane insulin 26 units daily, with an HbA1c of 38 mmol/mol (Fig. 3). She delivered a healthy infant weighing 4.2 kg via elective caesarean section. Breastfeeding was initiated after delivery. However, baby developed mild hypoglycaemia (2.5 mmol/L) six hours later, which improved with treatment.

### Case 2

**Figure 4:**
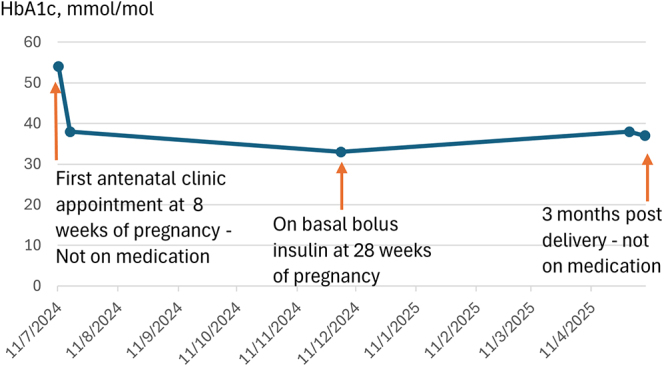
HbA1c trend in patient 2. The graph displays serial HbA1c measurements for patient 2, showing glycaemic control before, during and after pregnancy.

She was initiated on Lispro 4 units with meals and isophane insulin 4 units daily, alongside dietetic review. She was monitored fortnightly, and towards the end of pregnancy, her insulin requirements increased to Lispro 10 units with meals and isophane insulin 18 units daily. At 34 weeks of gestation, ultrasound assessment showed a fetal abdominal circumference at the 97th centile, with an HbA1c of 33 mmol/mol (Fig. 4). She delivered a healthy infant at 38 weeks of gestation, weighing 4.0 kg. The infant developed mild hypoglycaemia (2.6 mmol/L), which improved with treatment.

## Outcome and follow-up

### Case 1

Three months postpartum, she was only on only Lispro 8 units with meals, without isophane insulin, and was not breastfeeding. At that point, insulin was discontinued and tirzepatide was started, given her BMI of 40.3 kg/m^2^ and documented intolerance to both metformin and dapagliflozin. Her HbA1c and weight improved significantly with tirzepatide dose titration, and she is currently maintained on 10 mg weekly. Her most recent HbA1c is 32 mmol/mol, and BMI has improved to 32 kg/m^2^.

### Case 2

Insulin was discontinued postpartum, and she did not require any glucose-lowering medications. At her 3-month review, her HbA1c was 37 mmol/mol while managed with dietary modification alone. However, her BMI is 42 kg/m^2^, and she is scheduled for follow-up in our diabetes clinic in six months to consider initiating tirzepatide if her HbA1c increases.

## Discussion

Homozygous or compound heterozygous *RFX6* mutations cause Mitchell–Riley syndrome, an autosomal recessive disorder. It is characterized by pancreatic hypoplasia with neonatal hyperglycaemia, gallbladder hypoplasia and intestinal atresia. Meanwhile, heterozygous protein-truncating variants of *RFX6* are associated with MODY. Clinical features and management differ between MODY types. Hence, understanding the pathophysiology of *RFX6*-MODY is important to explain its clinical manifestations. We report two patients with *RFX6*-MODY diagnosed during pregnancy.

MODY requires a single mutated gene for patients to develop diabetes. Hence, offspring of individuals with *RFX6*-MODY have a 50% chance of inheriting the mutation. However, not all carriers of the mutated *RFX6* gene develop diabetes by age 50, as penetrance is reduced compared with other MODY variants (5). The penetrance of *RFX6*-MODY is 27% by age 25 and 78% by age 51, compared with common MODY forms (*HNF1A* and *HNF4A*), which have 91–99% penetrance by age 50.

Initial phenotypic data published in 2017 suggested that *RFX6*-MODY presents later in life than other MODY forms (median age at diagnosis: 32 years; IQR: 24–46 years; *n* = 27) (5). However, additional cases have since been reported, with many patients diagnosed earlier, between ages 6 and 26 years (Table 1) (6, 7, 8). Hence, with increased genetic screening and diagnosis of *RFX6*-MODY, a more accurate penetrance estimate could be established.

**Table 1:** Summary of reported cases of RFX6-MODY from the literature and the current case series. The table compares clinical characteristics, treatment modalities and pregnancy-related considerations across previously published cases of RFX6-MODY and the two patients presented in this case series. Data include age at diabetes diagnosis, BMI, initial HbA1c, diabetes treatment outside of pregnancy and available pregnancy information.

Study/cases	PTS, *n*	Age at diagnosis (years)	BMI at diagnosis (kg/m^2^)	Initial HbA1c (mmol/mol)	Treatment in non-pregnant adults	Pregnancy consideration
Patel *et al.* (5)	27	32 (IQR: 24–46) (range: 13–64)	25.1 (IQR: 23–28)	51 (IQR: 45–70)	Diet: 15%; OHA: 54%; insulin: 19%; insulin + OHA: 12%	Limited. One of the carriers developed GDM
Artuso *et al.* (2)	3					Limited
Case 1		6	26	62	OHA (sitagliptin and metformin)	
Case 2		18	23.3	54	OHA (sitagliptin)	History of GDM and diagnosed with *RFX6*-MODY at the age of 43 years
Case 3		10	20.5	84	Insulin	
Akiba *et al.* (6)	3					Limited
Proband		10	15.1	76	Insulin	
Mother		26	24.5	>86	Diet	Diagnosed with diabetes during first pregnancy
Mat. grandmother		50	21.2	N/A	OHA (metformin + sulphonylurea)	
Imaki *et al.* (7)	3					Not reported
Proband		16	20.8	95	OHA (metformin and liraglutide)	
Brother		16	N/A	N/A	Insulin	
Mother		46	N/A	N/A	Diet	
Li M *et al.* (8)	1	13 years	20	120	Insulin	Not reported
Our case series	2					Two type 2 DM patients were diagnosed with *RFX6*-MODY during pregnancy. Diabetes progression and management during pregnancy have been described in the case series
Case 1		19	31.6	81	Diet	
Case 2		13	N/A*	N/A^†^	Diet	

OHA, oral hypoglycaemic agent; N/A, not available; Mat. grandmother, maternal grandmother; PTS, patients.

* The earliest BMI available is 43.5.

^†^ The earliest HbA1c available is 54 mmol/mol at the age of 23 years.

Neither of our patients required insulin at diagnosis. Both were initially managed with dietary control but required insulin during pregnancy. This is consistent with *RFX6*-MODY phenotype, where most patients do not initially require insulin, as evidenced by adequate C-peptide levels (5). This likely occurs because although mutated *RFX6* impairs islet cell differentiation and insulin production, significant insulin deficiency may take years to develop. Over time, as β-cell dysfunction progresses and insulin production declines, insulin therapy may become necessary.

In addition to its role in β-cell function, *RFX6* is highly expressed in pancreatic α-cells and is required for regulated glucagon secretion. Experimental suppression of *RFX6* in human islets impairs α-cell exocytosis and causes dysregulated glucagon release (9). Although α-cell function has not been studied in individuals with *RFX6*-MODY, these findings suggest α-cell dysregulation and inappropriate glucagon secretion may contribute to diabetes development or worsening in affected individuals.

*RFX6*-MODY is also associated with low glucose-dependent insulinotropic polypeptide (GIP) levels (5). GIP is essential for regulating glucose-dependent insulin secretion and suppressing inappropriate glucagon secretion during hyperglycaemia. This GIP reduction may contribute to diabetes development in individuals with *RFX6* mutation, alongside pancreatic β-cell dysfunction. Reduced GIP levels in *RFX6*-MODY result from decreased expression and secretion from enteroendocrine K cells in the duodenum and jejunum. Hence, it would be interesting to assess real-world experiences managing *RFX6*-MODY with GIP/GLP-1 receptor agonists, particularly in patients with preserved C-peptide levels.

Our first patient responded well to tirzepatide, with significant improvement in HbA1c. To our knowledge, this is the first reported case treating *RFX6*-MODY with tirzepatide. Tirzepatide is a dual agonist of the GIP and GLP-1 receptors. By activating both receptors, it exerts synergistic effects, improving glucose-dependent insulin secretion, reducing inappropriate glucagon secretion, delaying gastric emptying and enhancing satiety. Its efficacy is supported by high affinity for the GIP receptor, comparable to endogenous GIP. Although tirzepatide is licensed for type 2 DM rather than *RFX6*-MODY, there is a physiological rationale to try this drug. However, its use should remain consistent with manufacturer recommendations and precautions, including avoidance during pregnancy and caution during breastfeeding.

Patients with *RFX6*-MODY who are planning pregnancy should be managed similarly to those with type 2 DM. This includes preconception counselling, optimization of medications and HbA1c and folic acid supplementation. As with other diabetes forms, pregnancy in patients with *RFX6*-MODY is expected to increase insulin resistance due to placental hormones, such as human placental lactogen, human placental growth hormone and human chorionic gonadotropin. However, these patients may be at a higher hyperglycaemia risk than those with type 2 DM, due to reduced basal GIP levels.

GIP levels typically rise in normal pregnancy, possibly as a counter-regulatory response to the diabetogenic state. However, low basal GIP levels in *RFX6*-MODY may contribute to postprandial hyperglycaemia from impaired insulin secretion and inappropriate glucagon production after meals. Patients with low GIP levels have a sixfold increased risk of developing gestational diabetes mellitus (GDM) (10). This risk is further supported by data, showing that *RFX6* carriers are 79% more likely to develop GDM than non-carriers (3). Therefore, close monitoring is essential to ensure optimal glycaemic control and appropriate fetal growth, as was done in our patients.

Young patients should be evaluated for possible MODY during initial general or antenatal diabetes clinic follow-up. A thorough medical history, including family diabetes history, should be obtained. As the oral glucose tolerance test is typically performed at 24–28 weeks to diagnose GDM, HbA1c is less likely to be elevated then. Therefore, an elevated HbA1c during initial screening may indicate abnormal glucose metabolism in early pregnancy or pregestational diabetes. MODY probability can be assessed using the MODY probability calculator, incorporating age at diagnosis, current age, sex, BMI, HbA1c, diabetes treatment, time to insulin initiation, family history, ethnicity and associated clinical features. A probability >25% is considered appropriate to support referral for genetic testing in individuals not treated with insulin within 6 months of diagnosis, as was done in our two patients diagnosed after their first antenatal diabetes clinic visit, while a lower threshold of >10% is used in those requiring insulin therapy (11).

In conclusion, a thorough family history is essential for identifying patients with MODY. Early diagnosis facilitates personalized treatment and may reduce the risk of developing both microvascular and macrovascular complications. Tirzepatide may be a useful therapeutic option in managing *RFX6*-MODY, as demonstrated by our patient’s favourable clinical response.

## Declaration of interest

The authors declare that there is no conflict of interest that could be perceived as prejudicing the impartiality of the study reported.

## Funding

This study did not receive any specific grant from any funding agency in the public, commercial or not-for-profit sector.

## Patient consent

Written informed consent was obtained from both patients for publication of their clinical details.

## Author contribution statement

PPG, RPN, SJM, RM, RS and SB are involved in the management of both patients. PPG drafted the first draft of the manuscript. All authors made individual contributions to authorship. All authors reviewed and approved the final draft.
